# Cellular irinotecan resistance in colorectal cancer and overcoming irinotecan refractoriness through various combination trials including DNA methyltransferase inhibitors: a review

**DOI:** 10.20517/cdr.2021.82

**Published:** 2021-11-02

**Authors:** Shogo Ozawa, Toshitaka Miura, Jun Terashima, Wataru Habano

**Affiliations:** Division of Pharmacodynamics and Molecular Genetics, School of Pharmacy, Iwate Medical University, Yahaba, Iwate 028-3694, Japan.

**Keywords:** Drug resistance, colorectal cancer, anti-cancer drugs, irinotecan, ABCG2, DNA topoisomerase I, cancer stem cells, epigenetics

## Abstract

Treatment with pharmacological drugs for colorectal cancer (CRC) remains unsatisfactory. A major cause of failure in pharmacotherapy is the resistance of colon cancer cells to the drugs, creating an urgent issue. In this review, we summarize previous studies on the resistance of CRC cells to irinotecan and discuss possible reasons for refractoriness. Our review presents the following five major causes of irinotecan resistance in human CRC: (1) cellular irinotecan resistance is induced mainly through the increased expression of the drug efflux transporter, *ABCG2*; (2) cellular irinotecan resistance is also induced in association with a nuclear receptor, pregnane/steroid X receptor (PXR/SXR), which is enriched in the *CYP3A4* gene enhancer region in CRC cells by exposing the cells to SN-38; (3) irinotecan-resistant cells possess either reduced DNA topoisomerase I (Top1) expression at both the mRNA and protein levels or Top1 missense mutations; (4) alterations in the tumor microenvironment lead to drug resistance through intercellular vesicle-mediated transmission of miRNAs; and (5) CRC stem cells are the most difficult targets to successfully treat CRC. In the clinical setting, CRC gradually develops resistance to initially effective irinotecan-based therapy. To solve this problem, several clinical trials, such as irinotecan plus cetuximab *vs.* cetuximab monotherapy, have been conducted. Another clinical trial on irinotecan plus guadecitabine, a DNA-methyltransferase inhibitor, has also been conducted.

## INTRODUCTION

**Figure 1:**
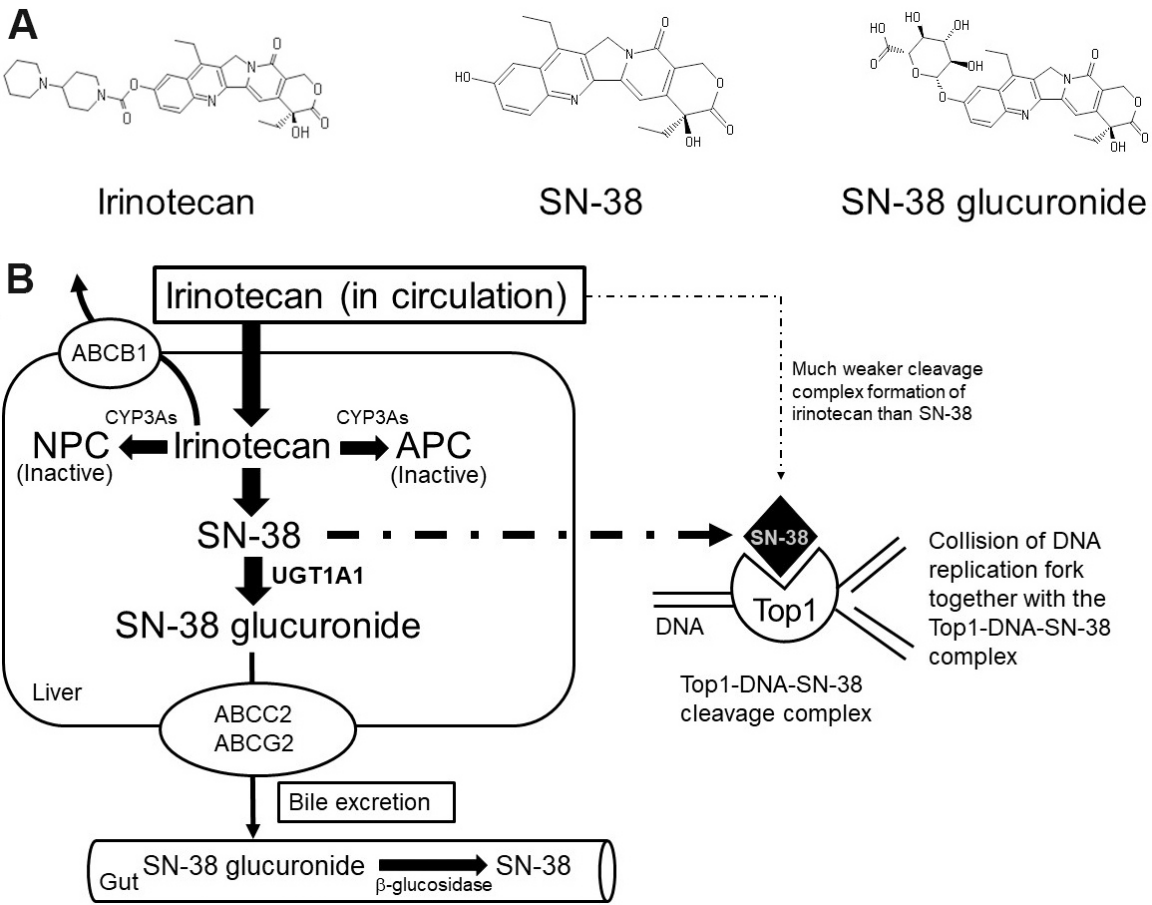
Irinotecan, the active metabolite, SN-38, and its glucuronide (A), and formation of inhibitory complex of Top1-DNA-SN-38 much more potent than irinotecan (B). In liver, irinotecan is bioactivated through hydrolysis catalyzed by carboxylesterases to be converted into SN-38, an active metabolite of irinotecan. SN-38 undergoes glucuronide conjugation to be SN-38 glucuronide by a UDP-glucuronosyltransferase, UGT1A1, for the detoxification pathway. SN-38 glucuronide is hydrolyzed by b-glucuronidase after being excreted in the gut. In the tumor cells, SN-38 can target the Top1-DNA covalent reaction intermediates and reversibly stabilize the Top1-DNA-SN-38 complex. As illustrated in (B), the collision of the DNA replication fork, together with this ternary complex formation, ultimately results in lethal and irreversible double-strand breaks^[4]^. Top1: Topoisomerase I.

Irinotecan (irinotecan hydrochloride hydrate) was developed and approved in Japan in 1994 as the first camptothecin derivative anti-cancer drug^[1]^. The contribution of irinotecan to treat a wide variety of advanced solid tumors was extensively reviewed in 2019^[2]^. The review article describes useful regimens of drug combinations, such as folinic acid, 5-fluorouracil (5-FU), and irinotecan (FOLFIRI) and folinic acid, 5-FU, irinotecan, and oxiplatin (FOLFIRINOX), to treat metastatic or advanced solid cancers. In contrast, post-treatment surveillance of patients treated with irinotecan in 2011 in Japan reconfirmed the serious incidences of leukopenia, thrombocytopenia, and diarrhea^[3]^. Irinotecan is a prodrug that is bioactivated through hydrolysis catalyzed by carboxylesterases to be converted to SN-38 in the liver. SN-38 itself is a very active anti-cancer metabolite of irinotecan that undergoes glucuronide conjugation by a UDP-glucuronosyltransferase, UGT1A1, for the detoxification pathway [Figure 1]. SN-38 glucuronide is hydrolyzed by b-glucuronidase after being excreted in the gut. In the tumor cells, SN-38 can target topoisomerase I (Top1)-DNA covalent reaction intermediates and reversibly stabilize the Top1-DNA-SN-38 complex. As illustrated in Figure 1B, the collision of the DNA replication fork, together with this ternary complex formation, ultimately results in lethal and irreversible double-strand breaks^[4]^. Individual differences in metabolic capacities should thus be considered for individualized irinotecan therapy^[5]^. The mechanism of the anti-cancer activity of SN-38 is the stabilization of the DNA Top1-DNA cleavable complex in cancer cells, resulting in the arrest of DNA replication^[2]^.

Even though various human cancers are treated with irinotecan, refractoriness remains a significant problem, as is the case with most other anti-cancer drugs. With regard to drug therapy for colorectal cancer (CRC), cellular and molecular mechanisms of failure in irinotecan therapy seem to be roughly classified into the following six categories: (1) drug uptake and export; (2) drug metabolism; (3) changes in drug targets; (4) phenotype transition; (5) adaptation to the tumor microenvironment; and (6) colon cancer stem cells (CSCs)^[6]^.

Recently, epigenetic alterations have attracted attention with relation to CRC phenotypes and sensitization of cancer cells by combining epigenetic modifying agents with existing anti-cancer agents^[7,8]^.

In this article, we review studies on irinotecan resistance, along with clinical issues that are likely to reduce the therapeutic effectiveness of irinotecan. We provide insights into various clinical trials to overcome irinotecan refractoriness including a combination treatment of DNA-methyltransferase inhibitors with irinotecan.

## MECHANISMS OF IRINOTECAN REFRACTORINESS

Mechanisms of irinotecan refractoriness could be overexpression of drug efflux transporters, elevated drug metabolism for detoxification, and changes in Top1, cancer cell phenotypes, and tumor microenvironment. Furthermore, we describe CSCs causing failure in chemotherapy.

### Changes in the expression levels of drug efflux transporters in human colon cancer cells

**Figure 2:**
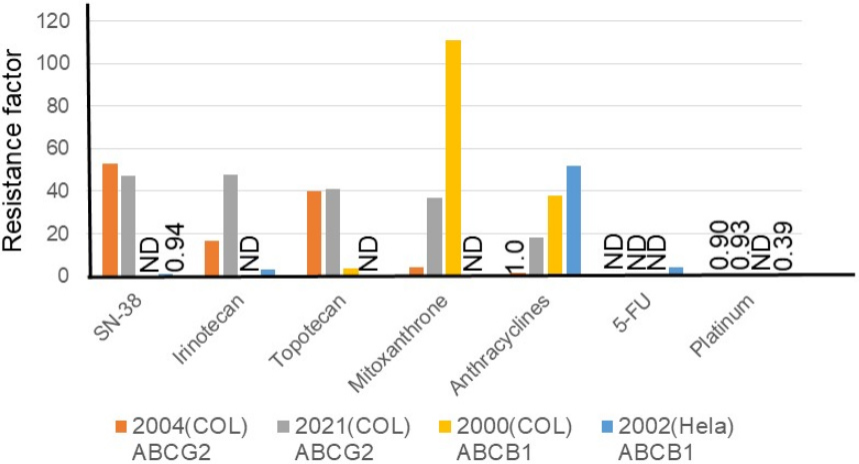
Factors causing resistance to anti-cancer drugs in ABCB1- or ABCG2-overexpressing colon cancer cell lines and an ABCB1-overexpressing HeLa cell line. Resistance factors for SN-38, irinotecan, topotecan, mitoxantrone, anthracyclines, 5-fluorouracil, and platinum anticancer drugs are shown relative to the respective parental cell lines. Cell lines are HCT116-SN6 [2004 (COL), ABCG2-overexpressing colon cancer cell^[10]^ (orange)], S1-IR20 [2021 (COL), ABCG2-overexpressing colon cancer cell^[11]^ (gray)], S1-B1-20 [2000 (COL), ABCB1-overexpressing colon cancer cell^[12]^ (yellow)], and Hvr100-6 [2002 (HeLa), ABCB1-overexpressing HeLa cell^[13]^ (blue)]. Establishments of these resistant sublines are described in Table 1. ND: Not determined.

Overexpression of ATP-binding cassette drug efflux transporters (ABC transporters, such as ABCB1 or MDR1) on the plasma membrane of cancer cell lines, isolated as paclitaxel- or olaparib-resistant cells, has been regarded as a mechanism of drug resistance toward relatively lipophilic anti-cancer drugs, such as doxorubicin, rucaparib, paclitaxel, and olaparib, for a long time^[9]^. Overexpression of a second ABC transporter, ABCG2 [breast cancer resistance protein (BCRP)], is more significant in irinotecan resistance^[10-13]^. Different research groups have independently established ABCB1-^[12,13]^ and/or ABCG2-overexpressing^[10-12] ^cancer cell lines. As illustrated in Figure 2, higher resistance factors were observed for SN-38 (approximately 50-fold), irinotecan (17-48-fold), and topotecan (approximately 40-fold) in ABCG2-overexpressing cells compared to ABCB1-overexpressing cells. On the other hand, an increased level of resistance was observed for anthracyclines (38-52-fold) in ABCB1-overexpressing cells compared to ABCG2-overexpressing cells. ABCG2 cDNA-mediated expression in human osteosarcoma Saos-2 cells made them highly resistant (approximately 100-fold) to irinotecan and SN-38^[14]^. Maliepaard *et al.*^[15]^ established a topotecan-selected and topotecan-resistant human ovarian cancer cell line, T8, which showed 176-fold resistance to SN-38 and 11-fold resistance to mitoxantrone from the human IGROV1 ovarian cancer cell line as the parental cell. They also demonstrated that the resistance was due to increased topotecan efflux, which led to decreased intracellular accumulation of the drug^[15]^. The drug-resistant T8 subline was shown to overexpress the BCRP/mitoxantrone resistance/placenta-specific ABC transporter (*BCRP/MXR/ABCP*) gene^[15]^. Owatari *et al.*^[16]^ reported that copper-transporting P-type ATPase, ATP7A, confers drug resistance to SN-38, irinotecan, paclitaxel, vincristine, doxorubicin, etoposide, and mitoxantrone by ATP7A cDNA-mediated expression in ovarian cells of a Chinese hamster and immortalized fibroblasts from a patient with Menkes disease.

**Table 1:** Characterization of established drug-resistant human cancer cell lines

**Cells **	**Methods for induction of drug resistance**	**Cross resistance**	**Mechanisms of drug resistance **	**Ref.**
Ovarian cancer A2780 Parental cells	Continuous incremental drug selection		No ABCB1 expression	Vaidyanathan *et al.*^[9]^
Olaparib resistant A2780 (A2780olapR)	Olaparib (1-20 μM) (37-fold resistant to olaparib)	No cross resistance to paclitaxel	Higher ABCB1 expression (≥ 10-fold)	
Paclitaxel resistant A2780 (A2780pacR)	Paclitaxel (3 nM-2 mM) (7-fold resistant to paclitaxel)	Cross-resistance to olaparib (37-fold)	Relative ABCB1 expression (≥ 50-fold)	
Colon cancer HCT116 Parental cells HCT116-s	Continuously exposed to SN-38 (1-15 nM)	HCT116-SN6 and -SN50 were resistant to irinotecan and topotecan	Growth rate was slower in HCT116-SN50 than in HCT116-SN6	Candeil *et al*.^[10]^
HCT116-SN6 HCT116-SN50	Growing in 10 nM SN-38 Growing in 15 nM SN-38	Only HCT116-SN50 was resistant to mitoxantrone and doxorubicin	Mechanism not identified Overexpression of ABCG2	
Colon cancer S1 (parental cells) S1-IR20 (irinotecan resistant, 48-fold)	S1-IR20 established after 3-5 cycles of exposure to 0.5 mM irinotecan	S1-IR20, cross-resistant to SN-38 (RR = 47), topotecan (41), and mitoxantrone (37)	Overexpression of ABCG2 protein, but not ABCB1 or ABCC1. No change in Top1 protein	Wu *et al*.^[11]^
Colon cancer S1 (parental cells) S1-B1-20 (bisantrene resistant) S1-M1-80 (mitoxantrone resistant)	S1-B1-20 by exposure to 20 mM bisantrene; S1-M1-80 by exposure to increasing concentrations of mitoxantrone	S1-B1-20 (RR) resistance: mitoxantrone (111), daunorubicin (38), vinblastine (167), paclitaxel (285), and topotecan (3) S1-M1-80 (RR): mitoxantrone (35,100), and topotecan (680)	Higher ABCB1 level (14-fold) in S1-B1-20 and higher ABCG2 level (> 42,000-fold) in S1-M1-80	Litman *et al*.^[12]^
HeLa cells (parental cells) Hvr1-1 cells: vinblastine (12) Hvr10-6 cells: vinblastine (166) Hvr100-6 cells: vinblastine (498)	HeLa cells exposed to vinblastine (1, 10, and 100 nM) in a stepwise manner	Hvr100-6 (RR): doxorubicin (52), vincristine (327), paclitaxel (4145) Not resistant to platinum derivatives	Higher level of ABCB1, but not ABCC1, in fluorescence-activated cell sorter analysis. ABCC1 and ABCG2 mRNA levels were comparable to those in HeLa cells	Takara *et al*.^[13]^
A bone osteosarcoma cell line, Saos-2 ABCG2 #4 clone	cDNA-mediated ABCG2 expression in Saos-2 cells	ABCG2#4 (RR) resistant to irinotecan (168) and SN-38 (18)	ABCG2	Wierdl *et al*.^[14]^
An ovarian carcinoma cell line, IFROV1 T8 cells (52-fold topotecan resistant) MX3 cells (11-fold mitoxantrone resistant)	The T8 cell line was developed by exposure to increasing topotecan concentrations (24-240 nM) The MX3 cell line survived after 72 h exposure to 170 nM mitoxantrone	T8 cells, resistant (RR) to SN-38 (176) and mitoxantrone (11) MX3 cells, resistant to topotecan (14) and SN-38 (44)	Overexpression of breast cancer resistance protein, or ABCG2	Maliepaard *et al*.^[15]^
Me32a-T22/2L: an immortalized fibroblast from a Menkes disease patient	Me32a/pCMB117: Me32a-T22/2L transfected with *ATP7A* cDNA	Me32a/pCMB117 was resistant to (RR) to SN-38 (43), irinotecan (13), paclitaxel (94), vincristine (70), and doxorubicin (13)	Overexpression of ATP7A protein in Me32a/pCMB117 (14) compared with Me32a-T22/2L	Owatari *et al*.^[16]^
Doxorubicin-resistant MCF-7 sublines	Resistant cells induced by low-dose doxorubicin Acetylated histone H3, RNA polymerase II was enriched to the proximal ABCG2 promoter region	Degree of doxorubicin resistance ranged 1.3-3.6-fold	Increased ABCG2 expression Epigenetic alteration was observed in the resistant sublines	Calcagno *et al*.^[17]^
Colon cancer S1-B1-80 cells	S1-M1-80 was established by maintenance in the presence of increasing concentrations of mitoxantrone	An HuR protein that binds to AU-rich mRNA elements by enhancing their stability. Low miR-519c expression is correlated with high HuR and ABCG2 expression	Increased ABCG2 expression Epigenetic mechanism is likely to be involved	To *et al*.^[18]^

RR: Relative resistance compared to wild type cells.

**Figure 3:**
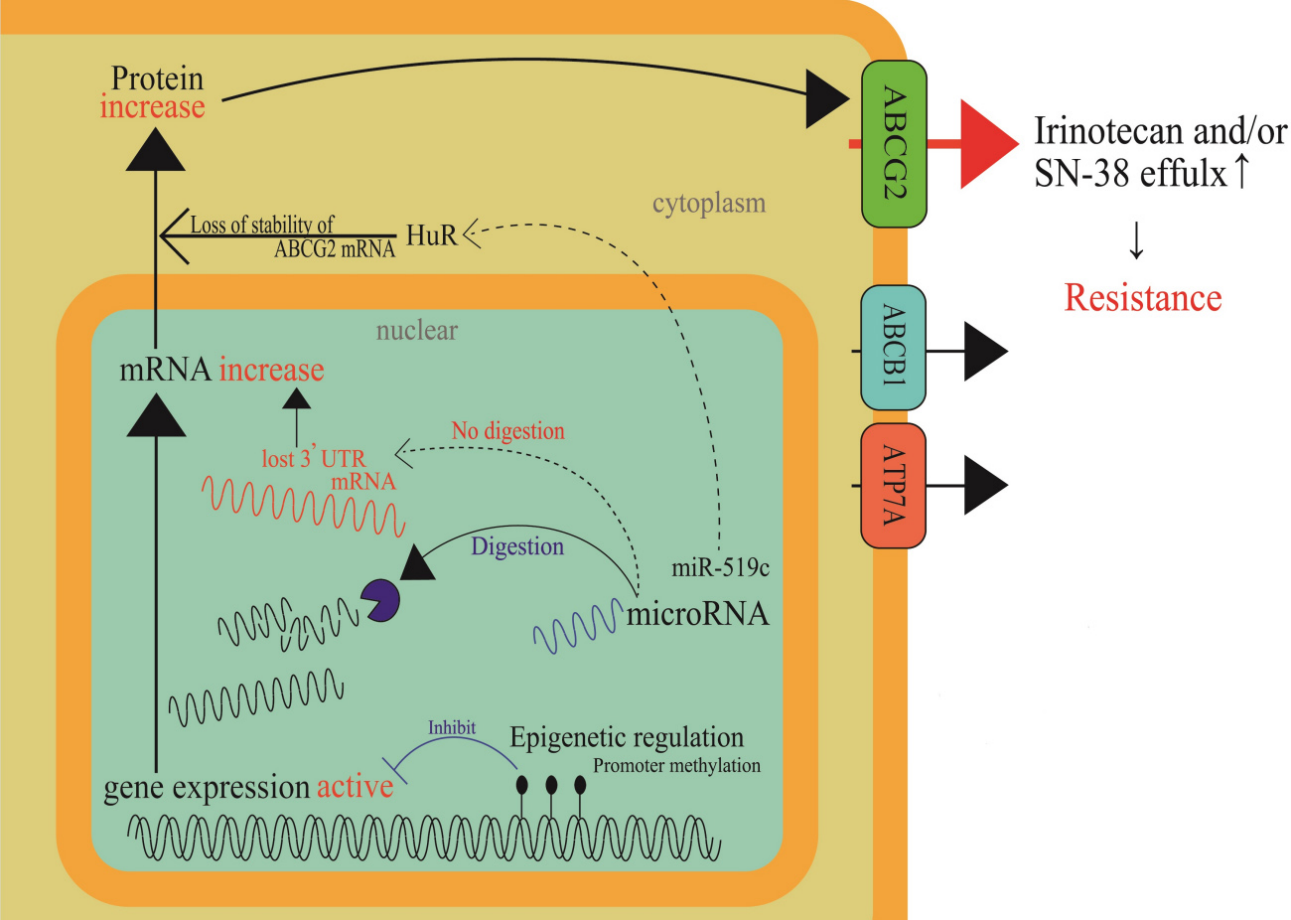
Epigenetic regulation mechanisms in cancer cells that result in acquired irinotecan resistance. Methylation in a promoter region of a drug efflux transporter, ABCG2, and stabilization of a mRNA binding protein, HuR, through miR-519c function are depicted as epigenetic regulation mechanisms. When positive regulation mechanisms are prevailing in cancer cells, *ABCG2* gene expression will be enhanced. Thus, cancer cells will become resistant to irinotecan.

Until recently, many studies have shown that ABC transporter expression is regulated by epigenetic modifications or microRNA (miRNA) regulation. Hypermethylation of the ABCG2 promoter decreased its mRNA levels. Demethylation by a DNA methyltransferase (DNMT) inhibitor was reported to recover mRNA expression and induce drug resistance^[19]^. H3K4me3 demethylation increases the expression of ABCC1 mRNA and protein^[20]^. Multidrug-resistant (MDR) sublines from MCF-7, IGROV1, and S1 cell lines were established from treatment with low doses of either etoposide or doxorubicin; thus, ABCG2 expression was increased in the resistant cells. Considerably higher enrichment (approximately 10-fold) of acetylated histone H3 and RNA polymerase II to the proximal ABCG2 promoter has been detected in the low-dose doxorubicin-selected MCF-7 cells (1.3-3.6-fold resistant to doxorubicin) than in the parental MCF-7 cells. In contrast, a lower association of histone deacetylase 1-ABCG2 promoter was detected in cells with low-dose doxorubicin-selected MCF-7 cells^[17]^. The expression levels of some miRNAs have also been reported to correlate with ABCG2 expression; miR-181a^[21]^, miR-328^[22]^, and miR-487a^[23]^ (breast) and miR-519c^[18]^ and miR-520h^[24]^ (colon) were base-paired to ABCG2 mRNA 3’-UTR and the binding silenced ABCG2 expression. Drug-resistant breast cancer cell lines showed low expression levels of these miRNAs. miR-519c, whose target site in the 3’-UTR existed only in the parental S1 cells, was thought to be involved in indirect regulation through an mRNA-binding protein, HuR, resulting in the loss of stability of the ABCG2 mRNA molecules in the parental cells^[24]^. ABCG2 overexpression in a drug-resistant subline, S1-M1-80, which is derived from the S-1 cell line, lacked a binding target site for miR-519c in its 3’-UTR sequence of ABCG2 mRNA (i.e., short ABCG2 3’-UTR) and is likely to result in high ABCG2 expression^[24]^. Clinically, tumors resected from 17 out of 26 CRC patients, who were responsive to 5-FU-based chemotherapy, showed remarkably lower ABCG2 levels than in adjacent normal tissues. In contrast, some unresponsive patients treated with 5-FU-based chemotherapy had comparable or even higher tumoral expression of ABCG2 than in the adjacent normal tissues. Most of the unresponsive patients exhibited a high expression of tumoral ABCG2 and HuR simultaneously^[18]^. Further clinical analyses revealed that tumors from three out of nine unresponsive patients carried a short ABCG2 3’-UTR in the mRNA, which prevented miR-519c-mediated repression and led to overexpression of ABCG2 mRNA^[18]^. Figure 3 shows these cellular events in a schematic diagram.

### Elevated cellular drug metabolism in relation to pregnane X receptor or steroid and xenobiotic receptor

**Figure 4:**
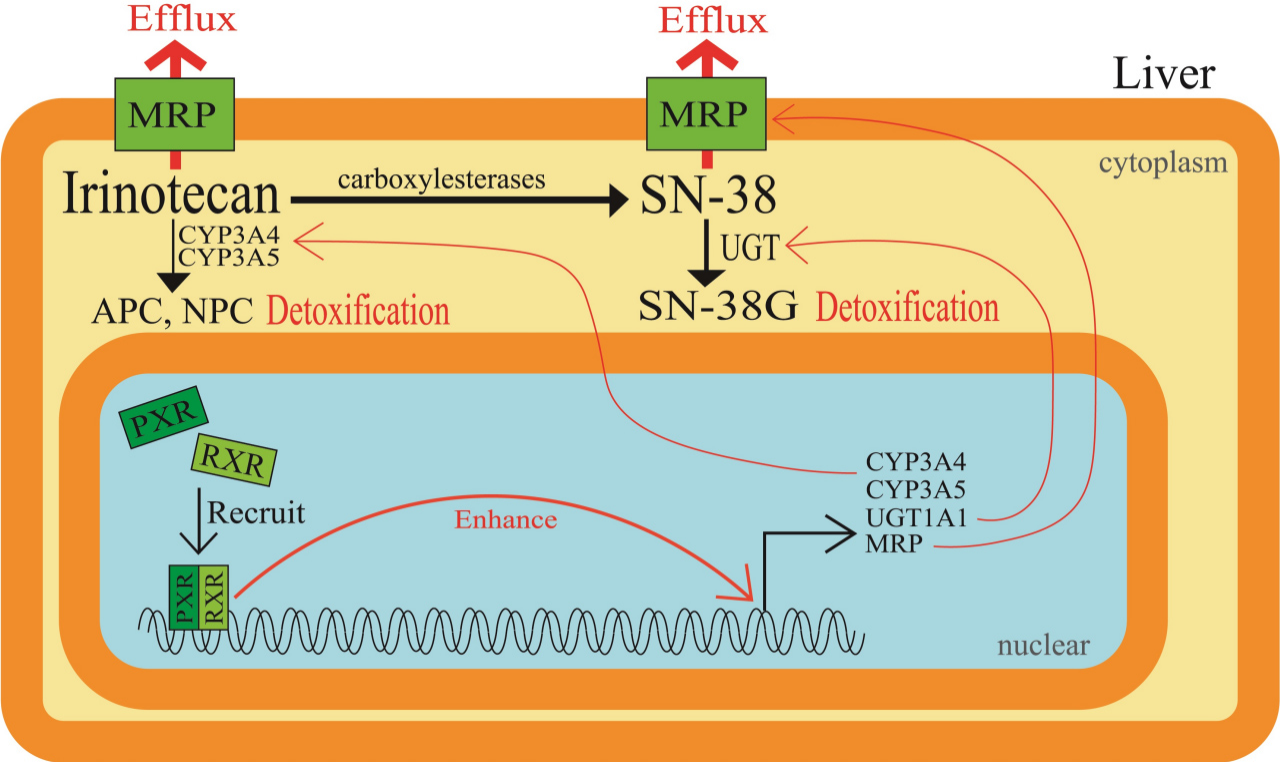
Elevated cellular drug metabolism in relation to pregnane X receptor or steroid and xenobiotic receptor. Induction of glucuronidation of SN-38 within colorectal cancer cells renders cells resistant to irinotecan-based therapy. PXR and SXR are well known as they are expressed mainly in mammalian livers and gastrointestinal tracts and are involved in the induction of various classes of drug-metabolizing enzymes and drug transporters to detoxify therapeutic drugs such as irinotecan and SN-38. PXR: Pregnane X receptor; SXR: steroid and xenobiotic receptor; RXR: retinoid X receptor.

It has been reported that irinotecan undergoes hepatic metabolism to be converted to CYP3A4-dependent oxidative metabolites, and it is then converted by carboxylesterases to an active metabolite SN-38. Next, the active SN-38 undergoes glucuronide conjugation to be an inactive metabolite, SN-38G. The induction of SN-38 glucuronidation within CRC cells renders the cancer cells resistant to irinotecan-based therapy. Pregnane X receptor (PXR) and steroid and xenobiotic receptor (SXR) are well-known since they are expressed mainly in mammalian liver and gastrointestinal tract. Moreover, they are involved in the induction of various classes of drug-metabolizing enzymes and drug transporters to detoxify xenobiotics and therapeutic drugs. cDNA-mediated expression of human PXR in cultured human colon cancer cells, LS174T, made the cells resistant to SN-38 by enhancing the glucuronidation of SN-38 to SN-38G. Conversely, suppression of PXR expression by short-hairpin RNA-expressing vectors decreased the intracellular and extracellular SN-38G/SN-38 ratios, in accordance with UGT1A1 downregulation^[25]^. Furthermore, SN-38 was shown to activate PXR in human colon cancer cell lines LS180 and HCT116 and induce CYP3A4, CYP3A5, UGT1A1, and the ABC transporter MRP2^[26]^. The levels of PXR and SXR expression are the highest in human livers, followed by intestinal tissue. Therefore, activation of PXR/SXR would result in the induction of various drug-metabolizing enzymes and drug transporters, all of which play a role in the disposition and excretion of irinotecan, SN-38, and SN-38G [Figure 4]. In fact, herb-irinotecan interactions have also been reviewed, where St. John’s wort is administered with anti-cancer drugs because it was suggested to exert mild to moderate effects against depression^[27]^. These results, together with the background studies on PXR/SXR, are summarized in Table 2.

**Table 2:** Elevation of cellular drug metabolism in relation to pregnane X receptor or steroid and xenobiotic receptor

**Background on PXR/SXR research**	**Materials and methods**	**Results and discussion**	**Ref.**
PXR can enhance detoxification of irinotecan in cancer cells	PXR cDNA-mediated expression in human colorectal cancer cell lines Human liver and colon biopsies	PXR cDNA-mediated overexpression led cell-resistance to irinotecan and SN38	Raynal *et al.*^[25]^
PXR/SXR is known to be involved in the upregulation of genes associated with the detoxification of irinotecan	ChIP with anti-PXR/SXR antibodies. PXR/SXR mediated induction of CYPs and ABC transporters after exposure of LS180 and HepG2 cells to irinotecan or SN-38	PXR enrichment induced by SN-38 treatment to *CYP3A4* gene enhancer and promoter regions SN-38 induced CYP3A4/5 (6-13-fold) and UGT1A1 and MRP2 by 2-3-fold. PXR/SXR decreased irinotecan cytotoxicity in LS180	Basseville *et al.*^[26]^

ChIP: Chromatin-immunoprecipitation; PXR: pregnane X receptor; SXR: steroid and xenobiotic receptor; CYPs: cytochrome P-450; ABC: ATP-binding cassette.

### Changes in drug targets

**Figure 5:**
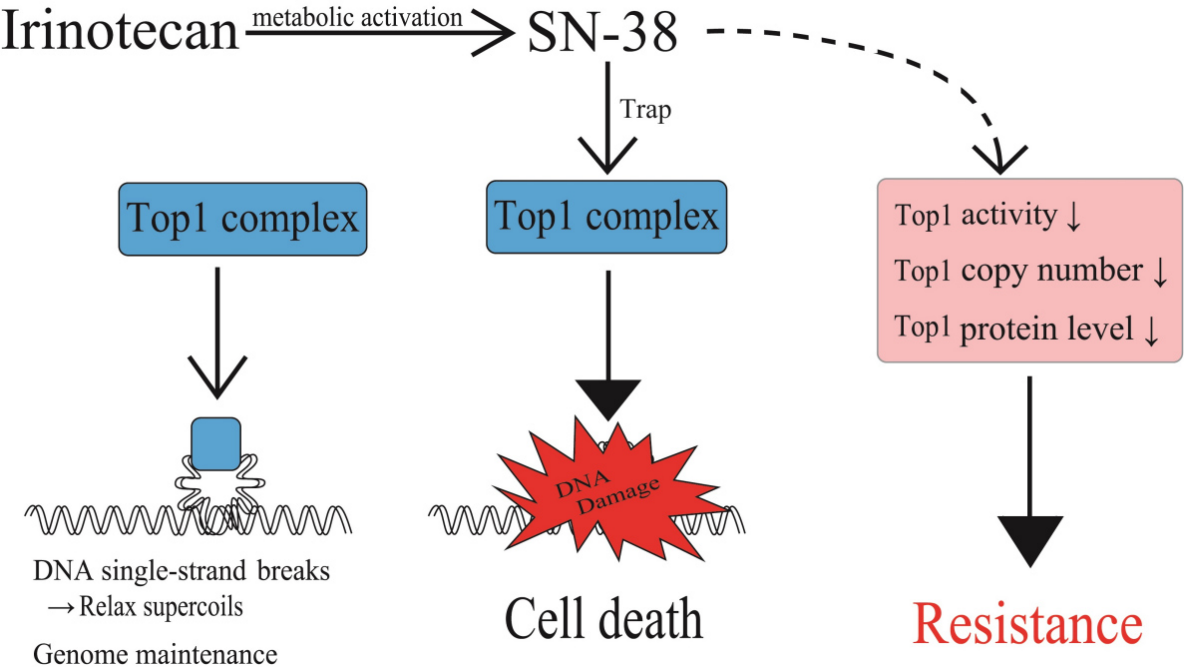
Factors affecting levels of the Top1-DNA cleavage complex and irinotecan resistance. Levels of irinotecan-causing DNA damage response depends upon formation of the Top1-DNA cleavage complex through Top1 function. Decreases in cellular Top1 activities, *Top1* gene copy number, and Top1 protein levels result in cellular irinotecan resistance. Top1: Topoisomerase I.

The molecular targets of the active metabolite of irinotecan, SN-38, are Top1-DNA covalent reaction intermediates that are reversibly stabilized by SN-38 and result in DNA strand breaks [Figure 5]^[28]^. DNA damage by irinotecan has been documented in regards to an increase in DNA damage by the inhibition of proteins, such as Rad51 and Rad52, which are involved in DNA repair. Curcumin was reported to target homologous recombination through the inhibition of Rad52, resulting in the sensitization of irinotecan-inducing DNA damage^[29]^. Flavopiridol, a cyclin-dependent kinase inhibitor, was shown to be beneficial to patients with advanced solid tumors that expressed wild-type p53 treated with irinotecan through the suppression of Rad51^[30]^. Resistance in Top1-targeting drugs has been attributed to qualitative and quantitative alterations of Top1. Since the properties of drug resistance vary widely, we summarize these research results in Table 3^[31-37]^. Generally, irinotecan-resistant cells showed lower Top1 activity or protein levels. Variable copy numbers of *Top1* gene were reported in five different cultured human cancer cells [Table 3]. The *Top1* gene locus in chromosome 20q has been shown to be apt to undergo chromosome alterations that result in gene dosage changes, as the locus is in proximity to an oncogene documented as “chromosome 20 deletions in myeloid malignancies”^[38]^.

**Table 3:** Changes in DNA topoisomerase I levels quantitatively or quantitatively affected cellular irinotecan sensitivity

**Cells**	**Change in sensitivity to irinotecan and structurally related Top1 inhibitors and relationship with changes in Top1 expression/primary structure**		**Ref.**
Human lung (A549), colon (HT29), stomach (St-4), and murine leukemia P388	The three human solid tumor cell lines were treated with a sublethal concentration of camptothecin and camptothecin-resistant P388 cells were established *in vivo* by treatment with 30 mg/kg of irinotecan over repeated transplantations	Top1 protein levels were significantly lower in resistant HT29/CPT, St-4/CPT, and P388/CPT, but not in resistant A549/CPT. Nuclear extracts (4.1 ng protein) from HT29 showed slightly higher Top1 activity than those (12.5 ng protein) from the resistant HT29/CPT cells. Resistance to topotecan is attributable to qualitative difference of Top1 proteins	Sugimoto *et al.*^[31]^
Human breast (MB MDA436, MB MDA231, ZR75-1, and MCF7) and a colon (HT29) cancer cell line	Change in *Top1* gene copy number: 1 and 4 in breast carcinoma cells, 5 in a breast carcinoma, 5 in a colon cancer; and 6 in a breast carcinoma	Cells with low *Top1* gene copy number show the highest resistance to SN-38 Copy number and Top1 protein expression highly correlated (*r* = 0.92)	McLeod and Keith^[32]^
A human colon cancer cell line, DLD-1	Missense Top1 mutation (Gly365Ser)	Cells exposed continuously to SN-38 were resistant to SN-38, camptothecin, and topotecan (10-100-fold) and carried a missense 365Ser allele	Arakawa *et al.*^[33]^
A human glioblastoma cell line, SF295	Two different camptothecin-resistant sublines were established by stepwise selection by BN80915 and homocamptothecin (both 50 nM)	Both sublines were 7-27-fold resistant to topotecan and camptothecin. Reduced Top1 mRNA (< 50%) and protein expressions were observed	Liao *et al*.^[34]^
A human colon cancer cell line, HCT116	Missense mutations of Top1 were found in 5 different resistant cell clones obtained by stepwise increased SN-38 concentrations: R621H, E710G and L617I	No difference in Top1 expression in the 5 resistant cells. All resistant cells treated with 1 mM SN-38 showed lower amounts of Top1-DNA complex as compared to the drug-sensitive HCT116	Gongora *et al.*^[35]^
Three human colon cancer cell lines, HCT116, HT29, and LoVo	Drug-resistant cells induced by exposing to increasing concentrations of SN-38 HCT116/SN-38 carried two Top1 mutations, R364K and G717R HT29/SN-38 showed 20% loss of Top1 compared with sensitive HT29 cells through chromosome 20q rearrangement	HT29/SN-38 and LoVo/SN-38 exhibited upregulations of ABCG2, by 25- and 60-fold, respectively. All SN-38-resistant cells were cross-resistant to an indenoisoquinoline non-camptothecin Top1-targeting drug in clinical trials	Jensen *et al.*^[36]^
Four human colon cancer cell-lines, HCT116, HT29, DLD1, and LoVo	HT29 cells express the least amount of carboxyl-terminal domain RNA polymerase II polypeptide small phosphatase 1 (CTDSP1) and were the most resistant to SN-38. HCT116 cells expressed CTDSP1 in a larger amount than HT29	siRNA-mediated downregulation of CTDSP1 in HCT116 cells activates the DNA-dependent protein kinase catalytic subunit (DNA-PKcs), which phosphorylated Top1 at Serine 10. The phosphorylated Top1 is apt to undergo SN-38-mediated proteasomal degradation. CTDSP1-downregulated HCT116 cells acquired a small degree of SN-38 resistance. HT29 cells showed a higher phosphorylation and activated status of DNA-PKcs. Top1 in HT29 cells was spontaneously and more easily degraded	Matsuoka *et al*.^[37]^

Top1: Topoisomerase I; CPT: camptothecin.

Copy number variations of the *Top1* gene were extensively investigated in normal mucosa and tumor tissue samples from 50 CRC patients. The analyses were performed using a fluorescence in situ hybridization probe mixture covering the *Top1* gene and a chromosome 20 centromere (CEN-20). The results show that 32 out of 50 CRC samples had a Top1/CEN-20 ratio higher than those observed in the unaffected colorectal mucosa, and that 42 out of 50 samples had increased *Top1* gene copy number^[39]^. Top1 has been reported as a predictive biomarker: irinotecan and oxaliplatin failed to treat patients with low Top1 but yielded major improvement in patients with moderate/high Top1^[40]^. Furthermore, the same clinical study indicated that patients with a high Top1 showed major improvement in overall survival (OS) with first-line combination chemotherapy (5-fluorouracil combined with irinotecan or oxaliplatin)^[40]^. It was reported that the germline mutation frequency in *Top1* gene was so low that it was not detected in 236 subjects or in 16 untreated lung cancer tissues. These results suggest that *Top1* gene mutation seldomly occurs in human subjects and human cancer tissues^[41]^.

### Transition of colon cancer cell phenotypes for acquiring irinotecan resistance

**Figure 6:**
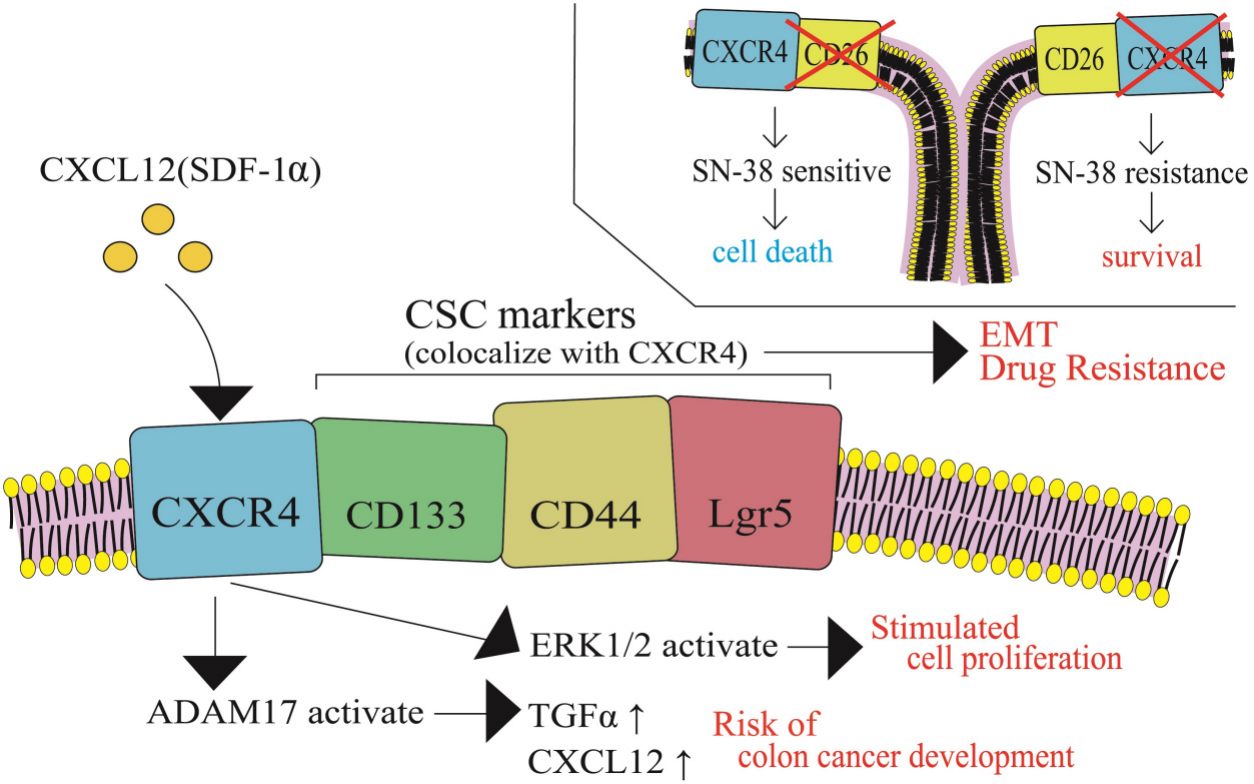
Transition of colon cancer cell phenotypes in relation to alteration of status of CXCR4 and CRC markers for acquiring irinotecan resistance. The G-protein coupled chemokine receptor, CXCR4, co-localizes with CRC stem cell markers, such as Lgr5, CD133, and CD44, which are thought to be associated with the epithelial-mesenchymal transition process and resistance to cancer drug therapy. CSC: Cancer stem cell; SDF-1α: stromal cell-derived factor-1α; TGFα: tumor growth factor-α; ERK1/2: extracellular signal-regulated kinase 1/2.

While irinotecan-resistant cancer cells are generally established by a stepwise dose escalation in an *in vitro* cell culture system, cancer cells *in vivo* are highly heterogeneous in CRC tumor mass. In this section, alterations in CRC cell phenotypes are discussed during cancer progression. A variety of biological factors may affect cancer cell phenotypes including cancer cell stemness, cell proliferation, metastasis, cell dormancy, and drug resistance. The G-protein coupled chemokine receptor, CXCR4, is highly expressed in CRC and has been shown to be associated with cancer stemness, metastasis, and poor prognosis. Furthermore, it has been shown that the CXCR4/CXCR7 pathway is activated when CXCL12 binds to either receptor^[42]^. In particular, CXCR4 receptor-ligand binding is thought to be associated with cancer stemness, development of metastasis, and poor prognosis. CXCR4 has been shown to co-localize with CRC stem cell markers, such as Lgr5, CD133, and CD44, which are thought to be associated with the epithelial-mesenchymal transition process and resistance to cancer drug therapy [Figure 6]^[42]^.

The expression of CXCR4 in colon cancer cells after treating with stromal cell-derived factor-1α (SDF-1α, or CXCL12) was investigated, along with the cancer cell proliferation rate and chemosensitivity to 5-FU, irinotecan, and oxaliplatin. This was carried out using high and low CXCR4-expressing human colon cancer HT29 sublines and SW480 cells with or without lentiviral CXCR4 overexpression^[43]^. Treatment with SDF-1α activated extracellular signal-regulated kinase 1/2 and stimulated cell proliferation. Higher expression levels of CXCR4 were associated with increased drug sensitivity to 5-FU, irinotecan, and oxaliplatin, compared to their low-CXCR4-expressing counterparts. Conversely, colon cancer cells with low CXCR4 levels were resistant to the tested anti-cancer agents, indicating that cancer cell dormancy is associated with drug resistance^[43]^. Another study showed that chemotherapeutic drugs, including SN-38, vinblastine, and methotrexate, decreased the population of CXCR4^+^/CD26^-^ cells and increased the CXCR4^-^/CD26^+^ cell population^[44]^. These two independent studies were consistent in that CXCR4^+^ cells were more sensitive to chemotherapeutic effects, and thus were preferentially eradicated; as a result, the CXCR4^-^ cell population became more prevalent. Gongora *et al.*^[45]^ also reported that a drug-resistant subline of human colon cancer, i.e., HCT116 cells (HCT116-SN6), which were 6-8-fold more resistant to SN-38, proliferated more slowly than the parental cell line (HCT116-s)^[10,45]^. Thus, tumor dormancy associated with CXCL12-CXCR4 signaling may be related to a drug-resistant phenotype in human colon cancer cells, which requires further investigation.

In addition, *A/J* mice who were fed a Western diet (WD, 20% fat; Normal, 5% fat) revealed WD-inducible metalloprotease (ADAM17) activation through CXCL12-CXCR4 signaling. ADAM17 activation, which is linked to the activation of epidermal growth factor receptors, increases CXCL12 in stromal cells and tumor growth factor-α (TGF-α) in colonocytes. This increase in the levels of CXCL12 and TGF-α is associated with an increased risk of colon cancer development^[46]^.

### The tumor microenvironment may affect cancer cell properties and drug sensitivity

**Figure 7:**
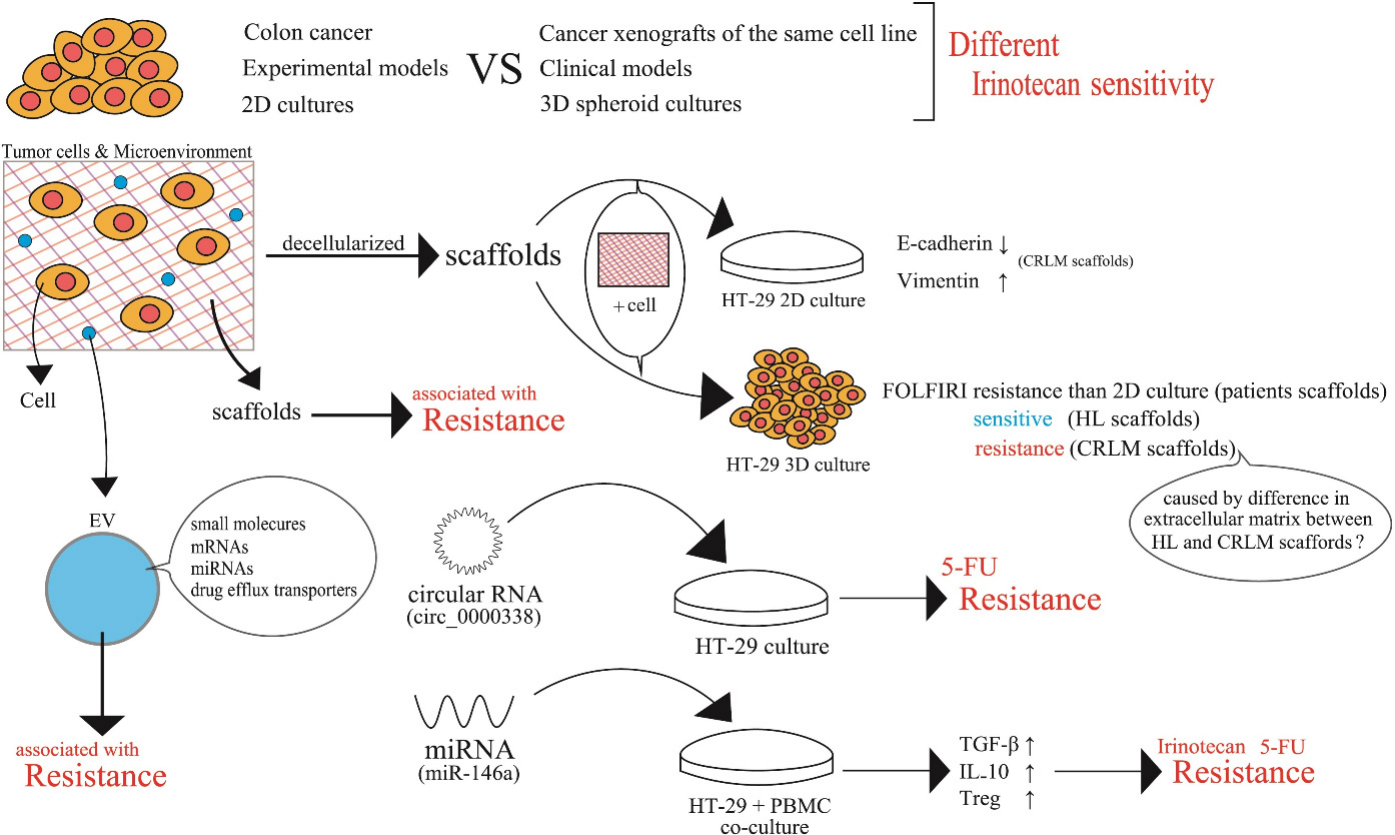
The tumor microenvironment may affect cancer cell properties and drug sensitivity/resistance. Scaffolds of cancer tissues were prepared by decellularization. Human colorectal cancer cells, HT-29, were inoculated on the resultant scaffolds to form 2D or 3D cultures of HT-29 cells. HT-29 cells in the 3D culture systems were created using scaffolds from healthy liver or colorectal cancer liver metastasis tissue, which showed overall resistance compared to 2D-cultured HT-29 cells. 3D-HT-29 cells on CRLM scaffolds were resistant to 5-fluorouracil. PBMC: Peripheral blood mononuclear cell; CRLM: colorectal cancer liver metastasis; HL: healthy liver; 5-FU: 5-fluorouracil; TGF-β: transforming growth factor-β; IL-10: interleukin-10; Treg: regulatory T cell.

There are many important findings on acquired cellular irinotecan resistance with enhanced efflux transporter activities, as well as the qualitative and quantitative changes in Top1. Irinotecan sensitivity or effectiveness might differ between a certain human colon cancer cell line and cancer xenografts of the same cell line, which might also be different between clinical CRCs consisting of cells with similar properties, even if pharmacokinetically equivalent irinotecan exposure was established between these experimental and clinical models. We reported that human liver cancer cell lines undergo differential gene regulatory mechanisms in two-dimensional (2D) plastic plate cultures and three-dimensional (3D) spheroid cultures^[47]^. In 2D culture, a nuclear receptor, aryl hydrocarbon receptor (AhR), mainly regulates CYP1A1 and CYP1A2. In 3D cultures, AhR mainly regulates CYP1A1, but not CYP1A2. Instead, PXR is involved in the regulation of CYP1A1, CYP1A2, or both, depending on the human liver cancer cell line^[47]^. Thus, the tumor microenvironment is very likely to affect gene regulatory mechanisms and other cellular functions. Our 3D culture systems seem to be extremely useful as a research tool for studying the tumor microenvironment in CRC cell lines. One important point to be considered is the difference in extracellular environments around individual cancer cells. D’Angelo *et al.*^[48]^ created new tissue culture models to study colorectal cancer liver metastasis (CRLM) by recruiting 18 CRLM and 5 CRC patients. The investigators conducted decellularization of healthy colon (HC), CRC, healthy liver (HL), and CRLM samples. They conducted Masson’s trichrome staining before and after decellularization. Human CRC HT-29 cells were cultured in patient-derived decellularized scaffolds derived from CRC and CRLM samples. Both scaffolds supported cell viability. In colorectal tissues, HT-29 cell viability was better in CRC than in HC samples. Investigation of the epithelial-mesenchymal transition profile revealed a significant reduction in E-cadherin expression and a significant increase in vimentin expression when HT-29 cells were cultured in CRLM scaffolds compared to HC, HL, and CRC. In the case of HCT116 cell culture in CRC and CRLM samples, E-cadherin and vimentin were expressed without any remarkable difference in HC, HL, CRC, or CRLM. Importantly, the 3D environment created with the patient-derived scaffolds showed a tendency to reduce the cellular sensitivity to 5-FU and FOLFIRI in comparison to those in 2D environments. Nevertheless, 3D-cultured HT-29 cells in HL scaffolds resulted in a significant reduction in cell proliferation following FOLFIRI treatment. The cytotoxic effects of FOLFIRI were not remarkable in HT-29 cells in a 3D CRLM scaffold culture. Cellular drug sensitivities influenced by the tumor microenvironment seem to vary between different cancer cells and anti-cancer drugs [Figure 7].

Another example of the tumor microenvironment association with drug resistance is extracellular vehicles (EVs), which mediate intercellular communication between cancer cells. EVs can contain small molecules such as therapeutic drugs, mRNAs, miRNAs, or even proteins, such as drug efflux transporters^[49,50]^. Exosome-mediated transfer of circular RNA (circ_0000338) enhanced 5-FU resistance in circular RNA-transmitted human CRC cell lines, which was achieved through its direct binding with miR-217 and miR-485-3p. Serum concentrations of circ_0000338 were higher in CRC patients who were resistant to the 5-FU regimen, which is consistent with the results of* in vitro* exosome-mediated circ_0000338 transfer [Figure 7]^[51]^.

Recent investigations have also shown that the expression of some miRNAs in colon cancer HT-29 cells may alter the colon cancer microenvironment and cell-to-cell interaction. miR-146a was overexpressed in HT-29 cells and co-cultured with peripheral blood mononuclear cells (PBMCs). miR-146a overexpression increased transforming growth factor-β and interleukin-10 expression in PBMCs. In a co-culture system of miR-146a, overexpressing HT-29 cells with PBMC enhanced the population of regulatory T cells (Tregs) and induced irinotecan and 5-FU resistance in HT-29 cells^[52]^. The underlying mechanisms of an increased population of Tregs and drug resistance in HT-29 cells remain to be determined.

The effects of the tumor microenvironment on cancer cell properties are summarized in Table 4.

**Table 4:** Features of the tumor microenvironment that may affect cancer cell property and drug sensitivity

**Research background**	**Description of tumor microenvironment**	**Microenvironment related effect**	**Ref.**
New tissue culture model for research on colorectal cancer liver metastasis	Scaffolds from HC, CRC, HL, and CRLM were created by decellularization of the corresponding tissues. HT-29 and HCT116 cells were kept three-dimensionally in the scaffold	Epithelial-mesenchymal transition was observed in HT-29 cell culture in CRLM scaffold. Two CRC cell lines, HT-29 and HCT116, were 3D cultured in four different scaffolds (HC, CRC, HL, and CRLM)	D’Angelo *et al.*^[48]^
Extracellular vesicles	EVs containing various biomolecules were created. The EV-containing biomolecules could be transferred to other cells	The biomolecules transferred to recipient cells alter the cellular phenotypes of the recipient cells	Fontana *et al*.^[49]^ Xavier* et al*.^[50]^
Exosome-mediated circular RNA transfer	Circ_000038 was transferred to recipient cells in an exosome-mediated fashion	The recipient cells became resistant to 5-fluorouracil by Circ_000038 with miR-217 and miR-485-3p	Zhao *et al*.^[51]^
A co-culture system of PBMCs and miR-146a-overexpressing HT-29 cells was created	miR-146a-overexpressing HT-29 cells and PBMC co-culture system led to increase in the population of regulatory T cell	Upregulated TNF-β and IL-10 in PBMCs and induction of irinotecan and 5-fluorouracil resistance were observed in miR-146a-overexpressing HT-29 cells	Khorrami *et al.*^[52]^

HC: Healthy colon; CRC: colorectal cancer; HL: healthy liver; CRLM: colorectal liver metastasis; EVs: extracellular vesicles; IL-10: interleukin-10; PBMCs: peripheral blood mononuclear cells; TNF-β: tumor necrosis factor-β.

### Colon cancer stem cells/colon cancer-initiating cells and drug-resistant phenotypes

Stem cells are defined as cells that can renew themselves, i.e., self-renew, and generate mature cells by differentiation to form their respective tissues. A well-known example is hematopoietic stem cells, which divide extensively in the bone marrow and are differentiated into common myeloid progenitors, erythrocytes, leukocytes, platelets, macrophages, and granulocytes, as well as into common lymphoid progenitors, B cells, T cells, natural killer cells, and dendritic cells. Stem cells often become targets of genetic events, resulting in malignant transformation. In abnormal processes, where signal transduction fails to function properly, stem cells may be dysregulated, leading to uncontrolled self-renewal and malignant transformation^[53]^. In colon cancer, Ricci-Vitiani *et al.*^[54]^ reported that tumorigenic cells in colon cancer existed as a high-density CD133^+^ population. The CD133^+^ cell population was present in approximately 2.5% of the total tumor cells. Tumorigenic CD133^+^ cells subcutaneously implanted in immunodeficient mice reproduced the original tumor properties. In contrast, CD133^-^ cells did not develop tumors^[54]^. Fang *et al.*^[55]^ reported that freshly isolated CD133^+^ colorectal adenocarcinoma cells from patients undergoing surgical resection were maintained for longer periods in tumor spheres, or so-called spheroids, and achieved long-term maintenance of CD133^+^ phenotype, retaining CSC properties. These isolated cells formed spheres under the adjusted medium for CSC culture. On the other hand, the cells could be induced to a differentiated state to lose CD133 expression in Dulbecco’s modified Eagle’s medium supplemented with 20% fetal bovine serum in collagen I-coated flasks. Importantly, treatment with irinotecan resulted in only 25% inhibition of clonogenicity of colon tumor spheroid cells, whereas 75% inhibition of clonogenicity of differentiated (adherent) population occurred. While more than 50% of the differentiated cells underwent 2 mM irinotecan-induced growth inhibition, 40% of the spheroid cells were resistant to irinotecan even at a concentration of 135 mM. These results clearly indicate that undifferentiated spheroid cells were more resistant to irinotecan than differentiated and adherent cells^[55]^. Altogether, in human colon cancer tissues, colon CSCs express CD133, a rare population of undifferentiated cells capable of self-renewal and maintaining the mass of colon cancer and drug-resistant phenotype.

**Figure 8:**
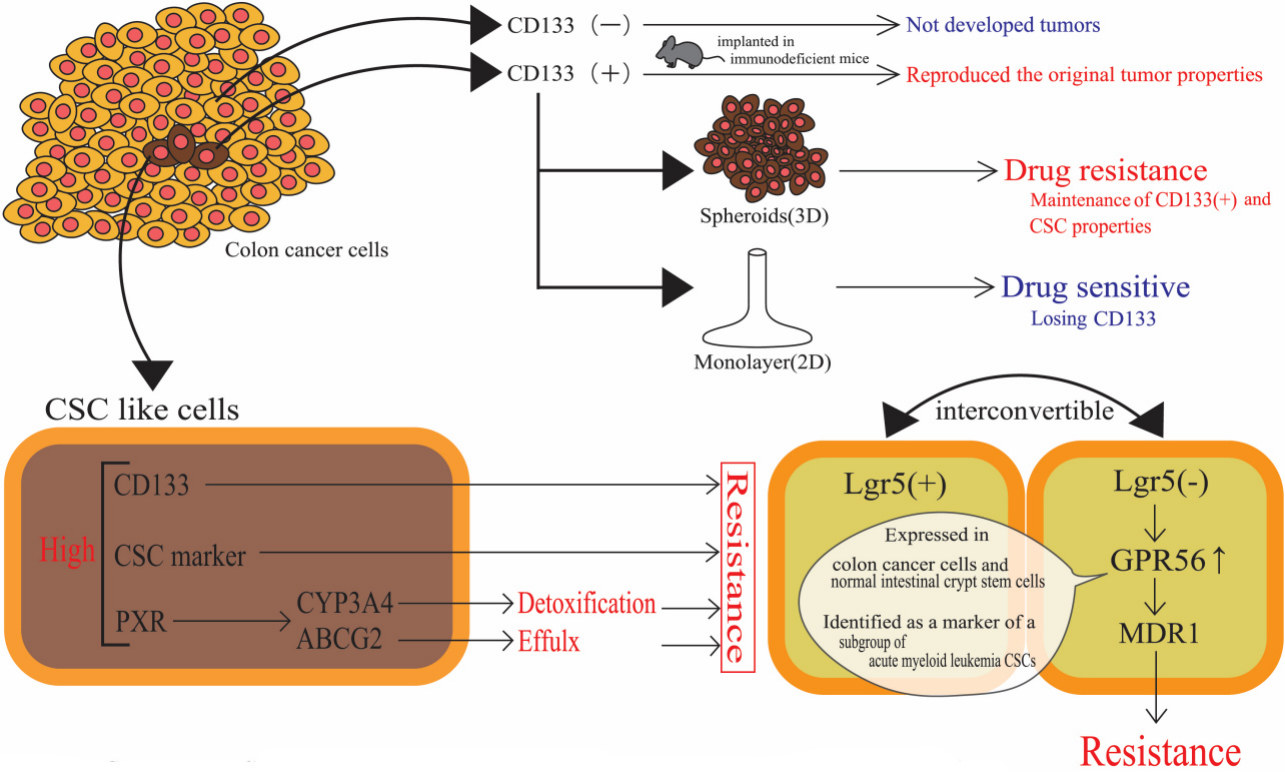
Cancer stem cells within a colorectal cancer tissue and drug resistance or drug sensitive phenotype. Tumorigenic cells in colon cancer existed as a high-density CD133^+^ population. Small population of CD133^+^ cells that are tumorigenic and drug resistant when subcutaneously implanted in immunodeficient mice reproduce the original tumor properties. In contrast, CD133^-^ cells do not develop tumors and show drug sensitive phenotype. CSC: Cancer stem cell.

Several investigators have reported drug-resistant phenotypes associated with increased activities or expressions of drug-metabolizing enzymes, drug transporters, and transcriptional regulators of gene products that function in drug metabolism and disposition. An *in vitro* colon CSC model was created by serum-free cell culture of human COLO 205 cells in a stem cell medium containing basic fibroblast growth factor, epidermal growth factor, and insulin-transferrin-selenite solution. Expression of CD133 was ascertained in CSC-like cells, and the CSC-like cells achieved approximately 15-, 10-, and 3.5-fold higher CD133, CYP3A4, and aldehyde dehydrogenase 1A1 (CSC marker) expression, respectively, compared to the control COLO 205 cells cultivated in standard cell medium (supplemented with 10% fetal bovine serum)^[56]^. The irinotecan-resistant phenotype emerged in the human colon cancer cell line LoVo, where a leucin-rich repeat-containing G-protein coupled receptor 5 (Lgr5) underwent siRNA-mediated knockdown. Lgr5 knockdown resulted in the expression of GPR56, a member of the adhesion G-protein coupled receptor subfamily. Reportedly, GPR56 is highly expressed in colon cancer cells. GPR56 has been shown to be expressed in normal intestinal crypt stem cells and has been identified as a marker of a subgroup of acute myeloid leukemia CSCs. Lgr5^+^ CRC cells and Lgr5^-^ cancer cells are known to be interconvertible. As already mentioned, Lgr5^-^ cells are regarded as resistant to irinotecan and 5-FU^[57]^. siRNA-mediated Lgr5 downregulation and GPR56 overexpression lead to increased expression of MDR1 (P-glycoprotein), which appears to be associated with irinotecan resistance^[57]^. Another study employed patient-derived CRC cells with CSC characteristics of self-renewal (i.e., forming spheroids) and resistance to chemotherapeutic agents. Patient-derived spheroids contained a higher proportion of cells with high aldehyde dehydrogenase (ALDH) activity, indicating CSC-like properties. Spheroidal cells showed a greater resistance (approximately 2-fold with statistical significance of *P *< 0.01, based on EC_50_) to a combination of 5-FU and SN-38, as compared to the same cells kept as adherent monolayers. Higher expression of PXR and colon CSC markers, including ALDH1A1, Oct-4, and Lgr5, along with PXR target genes (CYP3A4 and ABCG2), was observed in spheroids as compared with cells in 2D conditions [Figure 8]. Colon cancer cells resected from stage II and III patients showed a statistically significant positive correlation between PXR and ALDH1A1 mRNA levels. Moreover, stage II patients treated with 5-FU-based chemotherapy, who had tumors with higher PXR expression, showed poor prognosis^[58]^. These examples of CSC-associated drug resistance are summarized in Table 5.

**Table 5:** Cells representing “cancer stem cell” phenotype obtained from colorectal cancer patients

**Origin of CSC**	**Hallmarks of CSC**	**Drug resistance**	**Ref.**
Tumorigenic cells in colon cancer were isolated by mechanical and enzymatic dissociation. CD133 cells were separated magnetically or using a cell sorter	Tumorigenic cells in colon cancer were in the high-density CD133^+^ cells. CD133^+^ cells readily reproduced the original tumor in nude mice, but CD133^-^ cells did not form tumors	No description	Ricci-Vitiani *et al.*^[54]^
Colon cancer specimens were sliced and enzymatically dissociated. CSC cultures were made in a human embryonic stem cell medium with modification	Cell sorting was done on a MoFlo High Speed Cell Sorter using CD133 and EpCAM antibodies. Colon CSCs could be isolated and proliferate under serum-free and stem cell culture conditions. These spheroid forming cells express well-known CSC markers, such as CD133, CD166, CD44, and EpCAM	Relatively larger (> 70%) cell growth of spheroidal cells (showing CSC characteristics) was observed in irinotecan concentrations of 2 and 4 mM. Growth of differentiated cells (not showing CSC characteristics) at 2 and 4 mM was only 35%-40%. At 51 mM irinotecan, growth of spheroid cells and differentiated cells were approximately 35% and 10%, respectively	Fang *et al*.^[55]^
Human colon cancer cell line, COLO 205, cells were maintained in a medium free of serum	Culture under a serum-free condition for 7 days, and then CD133 expression was confirmed (CSC-like). The CSC-like cells express CYP3A4 and ALDH1A1 as compared with standard medium containing serum	CSC-like cells showed higher viability when exposed to oxaliplatin, cisplatin, gemcitabine, vinblastine, and etoposide	Olszewski *et al*.^[56]^
Patient-derived colorectal cancer cells (CRC1) maintained as spheroids	CRC1 cells maintained as spheroids showed a higher expression of ALDH activity, a CSC marker, than cells in 2D culture	Spheroidal cells were 2.44-fold more resistant to combinatory treatment of 5-fluorouracil and SN-38 than 2D cells	Planque *et al*.^[58]^

CSC: Cancer stem cell.

## CLINICAL PROBLEMS IN IRINOTECAN-BASED THERAPY AND OVERCOMING IRINOTECAN REFRACTORINESS THROUGH CLINICAL TRIALS INCLUDING DNA METHYLTRANSFERASE INHIBITORS

Since irinotecan was approved in 1994, it has been shown to be active in metastatic CRC. A randomized trial of irinotecan plus supportive care *vs.* supportive care alone was conducted in patients with metastatic CRC who were unresponsive to 5-fluorouracil treatment. The clinical trial showed that irinotecan treatment for patients with metastatic CRC after unresponsiveness to 5-fluorouracil treatment brought a longer survival and a better quality of life than patients with supportive care only^[59]^. However, refractoriness to irinotecan treatment gradually became a significant problem. To solve this issue, several clinical trials have been conducted: for example, studies on cetuximab monotherapy and cetuximab plus irinotecan in irinotecan-refractory metastatic colorectal cancer^[60]^; irinotecan plus cetuximab rechallenge^[61,62]^; trifluridine/tipiracil with or without bevacizumab^[63]^; regorafenib followed by cetuximab *vs.* the reverse sequence^[64]^; and FIRIS study^[65]^. The efficacy of cetuximab was reported as due to its pretreatment causing an increase (1.7-fold) in SN-38 AUCs in plasma and tumor of human colorectal carcinoma in xenograft mice, suggesting that cetuximab inhibits P-glycoprotein drug transporters^[66]^.

It has been shown that DNMT inhibitors increase mRNA and protein expression through the inhibition of chromatin condensation.

Several studies have reported that a combination of DNMT inhibitors, such as 5-aza-2’ deoxycytidine (DAC), with irinotecan could overcome irinotecan resistance by different mechanisms in human colon cancer cell models. We found that enhanced apoptosis by irinotecan was achieved in combination with DAC, through the downregulation of the anti-apoptotic gene Bcl-2^[67]^. Bcl-2/adenovirus E1B 19 kDa protein-interacting protein (BNIP3), a pro-apoptotic gene, was methylated in human colon cancer cell lines. BNIP3 upregulation led to the enhancement of irinotecan sensitivity^[68,69]^. Clinically, it has been suggested that BNIP3 methylation is associated with poor OS and shorter time to cancer progression^[69]^. Other researchers have reported glucocorticoid-induced protein-coding gene (DEXI) as an epigenetic marker of irinotecan resistance^[70]^. DEXI expression was suppressed in HCT116 cells by DNA methylation and was recovered by DAC exposure. In patients who underwent the FOLFIRI regimen, methylation-specific PCR analysis for a region including 11 CpG islands around exon 1 of *DEXI *revealed the association of methylated group patients with shorter progression-free survival and OS as compared to those with unmethylated *DEXI*; however, this association was not observed in patients who were administered the FOLFOX regimen. An increase in *DEXI* expression was likely to enhance apoptosis by irinotecan; however, the underlying mechanisms remain to be elucidated. *DEXI* reportedly functions as a modulator of virus-induced proinflammatory pathways in pancreatic β cells through the transcriptional activation of interferon-β^[71]^. Thus, demethylating agents of methylated DNA, such as azacytidine and decitabine, are thought to be clinically useful. Recently, a new DNMT inhibitor, guadecitabine, was developed and subjected to clinical trials in CRC patients previously exposed to irinotecan^[72]^.

## CONCLUSION

Our extensive review of the literature reveals the following five major issues leading to irinotecan resistance in human CRC:

(1) Cellular irinotecan resistance is induced mainly through the increased expressions of drug efflux transporters, ABCG2.

(2) Cellular irinotecan resistance is also induced in association with the nuclear receptor, PXR/SXR, which is enriched in the *CYP3A4* gene enhancer region in colon cancer cells by exposing the cells to SN-38.

(3) Irinotecan-resistant cells possess either reduced Top1 expression at both the mRNA and protein levels or Top1 missense mutations. Clinically, higher Top1 expression in CRC tissues can be a favorable predictive biomarker.

(4) Due to alterations in the tumor microenvironment, drug resistance occurs through intercellular vesicle-mediated transmission of miRNA.

(5) Colon CSCs, which reside in a small portion of the tumor mass and are highly tumorigenic and highly resistant to irinotecan and other anti-cancer drugs, are some of the most difficult targets for the successful treatment of colon cancer.

Although irinotecan is very useful and effective for CRC treatment in combination chemotherapy in FOLFIRI or FOLFIRINOX regimens, cellular irinotecan resistance often causes cancer relapse and metastasis. If irinotecan is considered as adjuvant chemotherapy for CRC, the expression of Top1, ABCG2, PXR/SXR, and CYP3A4 should be taken into consideration as possible causes of failure in drug treatment. Although difficult to achieve, monitoring changes in cancer cell phenotypes, including the expression of CD133, drug-metabolizing enzymes or drug transporters, Top1, and cell-to-cell communication-related genes, would be very useful for the efficacy of drug treatments of CRC. It would also be laudable to create spheroid cultures consisting of established human CRC-derived cell lines or primary colon cancer cells including generally drug-resistant colon CSC, which would be of great significance for basic and clinical research for drug treatments of CRC.

## DECLARATIONS

### Availability of data and materials

This review article was based on previously published research and reviews, which were collected by using a search of the literature, and they are cited in the review.

### Copyright

© The Author(s) 2021.

## References

[1] Sawada S, Okajima S, Aiyama R (1991). Synthesis and antitumor activity of 20(S)-camptothecin derivatives: carbamate-linked, water-soluble derivatives of 7-ethyl-10-hydroxycamptothecin. Chem Pharm Bull (Tokyo).

[2] Bailly C (2019). Irinotecan: 25 years of cancer treatment. Pharmacol Res.

[3] Tadokoro J, Kakihata K, Shimazaki M (2011). Post-marketing surveillance (PMS) of all patients treated with irinotecan in Japan: clinical experience and ADR profile of 13,935 patients. Jpn J Clin Oncol.

[4] Peters GJ

[5] (2018). Man FM, Goey AKL, van Schaik RHN, Mathijssen RHJ, Bins S. Individualization of irinotecan treatment: a review of pharmacokinetics, pharmacodynamics, and pharmacogenetics. Clin Pharmacokinet.

[6] Marin JJG, Macias RIR, Monte MJ (2020). Cellular mechanisms accounting for the refractoriness of colorectal carcinoma to pharmacological treatment. Cancers (Basel).

[7] Condelli V, Calice G, Cassano A (2021). Novel epigenetic eight-gene signature predictive of poor prognosis and MSI-like phenotype in human metastatic colorectal carcinomas. Cancers (Basel).

[8] Murai J, Thomas A, Miettinen M, Pommier Y (2019). Schlafen 11 (SLFN11), a restriction factor for replicative stress induced by DNA-targeting anti-cancer therapies. Pharmacol Ther.

[9] Vaidyanathan A, Sawers L, Gannon AL (2016). ABCB1 (MDR1) induction defines a common resistance mechanism in paclitaxel- and olaparib-resistant ovarian cancer cells. Br J Cancer.

[10] Candeil L, Gourdier I, Peyron D (2004). ABCG2 overexpression in colon cancer cells resistant to SN38 and in irinotecan-treated metastases. Int J Cancer.

[11] Wu ZX, Yang Y, Zeng L (2021). Establishment and characterization of an irinotecan-resistant human colon cancer cell line. Front Oncol.

[12] Litman T, Brangi M, Hudson E (2000). The multidrug-resistant phenotype associated with overexpression of the new ABC half-transporter, MXR (ABCG2). J Cell Sci.

[13] Takara K, Sakaeda T, Yagami T (2002). Cytotoxic effects of 27 anticancer drugs in HeLa and MDR1-overexpressing derivative cell lines. Biol Pharm Bull.

[14] Wierdl M, Wall A, Morton CL (2003). Carboxylesterase-mediated sensitization of human tumor cells to CPT-11 cannot override ABCG2-mediated drug resistance. Mol Pharmacol.

[15] Maliepaard M, van Gastelen MA, de Jong LA et al (1999). Overexpression of the BCRP/MXR/ABCP gene in a topotecan-selected ovarian tumor cell line. Cancer Res.

[16] Owatari S, Akune S, Komatsu M (2007). Copper-transporting P-type ATPase, ATP7A, confers multidrug resistance and its expression is related to resistance to SN-38 in clinical colon cancer. Cancer Res.

[17] Calcagno AM, Fostel JM, To KK (2008). Single-step doxorubicin-selected cancer cells overexpress the ABCG2 drug transporter through epigenetic changes. Br J Cancer.

[18] To KK, Leung WW, Ng SS (2015). Exploiting a novel miR-519c-HuR-ABCG2 regulatory pathway to overcome chemoresistance in colorectal cancer. Exp Cell Res.

[19] Moon HH, Kim SH, Ku JL (2016). Correlation between the promoter methylation status of ATP-binding cassette sub-family G member 2 and drug sensitivity in colorectal cancer cell lines. Oncol Rep.

[20] Lin H, Yang G, Yu J (2018). KDM5c inhibits multidrug resistance of colon cancer cell line by down-regulating ABCC1. Biomed Pharmacother.

[21] Jiao X, Zhao L, Ma M (2013). MiR-181a enhances drug sensitivity in mitoxantone-resistant breast cancer cells by targeting breast cancer resistance protein (BCRP/ABCG2). Breast Cancer Res Treat.

[22] Pan YZ, Morris ME, Yu AM (2009). MicroRNA-328 negatively regulates the expression of breast cancer resistance protein (BCRP/ABCG2) in human cancer cells. Mol Pharmacol.

[23] Ma MT, He M, Wang Y (2013). MiR-487a resensitizes mitoxantrone (MX)-resistant breast cancer cells (MCF-7/MX) to MX by targeting breast cancer resistance protein (BCRP/ABCG2). Cancer Lett.

[24] To KK, Zhan Z, Litman T, Bates SE (2008). Regulation of ABCG2 expression at the 3' untranslated region of its mRNA through modulation of transcript stability and protein translation by a putative microRNA in the S1 colon cancer cell line. Mol Cell Biol.

[25] Raynal C, Pascussi JM, Leguelinel G (2010). Pregnane X receptor (PXR) expression in colorectal cancer cells restricts irinotecan chemosensitivity through enhanced SN-38 glucuronidation. Mol Cancer.

[26] Basseville A, Preisser L, de Carné Trécesson S (2011). Irinotecan induces steroid and xenobiotic receptor (SXR) signaling to detoxification pathway in colon cancer cells. Mol Cancer.

[27] Meijerman I, Beijnen JH, Schellens JH (2006). Herb-drug interactions in oncology: focus on mechanisms of induction. Oncologist.

[28] Pommier Y, Sun Y, Huang SYN, Nitiss JL (2016). Roles of eukaryotic topoisomerases in transcription, replication and genomic stability. Nat Rev Mol Cell Biol.

[29] Tseng WC, Chen CY, Chern CY (2021). Targeting HR repair as a synthetic lethal approach to increase DNA damage sensitivity by a RAD52 inhibitor in BRCA2-deficient cancer cells. Int J Mol Sci.

[30] Ambrosini G, Seelman SL, Qin LX (2008). The cyclin-dependent kinase inhibitor flavopiridol potentiates the effects of topoisomerase I poisons by suppressing Rad51 expression in a p53-dependent manner. Cancer Res.

[31] Sugimoto Y, Tsukahara S, Oh-hara T, Isoe T, Tsuruo T (1990). Decreased expression of DNA topoisomerase I in camptothecin-resistant tumor cell lines as determined by a monoclonal antibody. Cancer Res.

[32] McLeod HL, Keith WN (1996). Variation in topoisomerase I gene copy number as a mechanism for intrinsic drug sensitivity. Br J Cancer.

[33] Arakawa Y, Suzuki H, Saito S, Yamada H (2006). Novel missense mutation of the DNA topoisomerase I gene in SN-38-resistant DLD-1 cells. Mol Cancer Ther.

[34] Liao Z, Robey RW, Guirouilh-Barbat J (2008). Reduced expression of DNA topoisomerase I in SF295 human glioblastoma cells selected for resistance to homocamptothecin and diflomotecan. Mol Pharmacol.

[35] Gongora C, Vezzio-Vie N, Tuduri S (2011). New topoisomerase I mutations are associated with resistance to camptothecin. Mol Cancer.

[36] Jensen NF, Agama K, Roy A (2016). Characterization of DNA topoisomerase I in three SN-38 resistant human colon cancer cell lines reveals a new pair of resistance-associated mutations. J Exp Clin Cancer Res.

[37] Matsuoka H, Ando K, Swayze EJ (2020). CTDSP1 inhibitor rabeprazole regulates DNA-PKcs dependent topoisomerase I degradation and irinotecan drug resistance in colorectal cancer. PLoS One.

[38] Bench AJ, Nacheva EP, Hood TL (2000). Chromosome 20 deletions in myeloid malignancies: reduction of the common deleted region, generation of a PAC/BAC contig and identification of candidate genes. UK Cancer Cytogenetics Group (UKCCG). Oncogene.

[39] Rømer MU, Jensen NF, Nielsen SL (2012). TOP1 gene copy numbers in colorectal cancer samples and cell lines and their association to in vitro drug sensitivity. Scand J Gastroenterol.

[40] Braun MS, Richman SD, Quirke P (2008). Predictive biomarkers of chemotherapy efficacy in colorectal cancer: results from the UK MRC FOCUS trial. J Clin Oncol.

[41] Fukui T, Mitsufuji H, Kubota M (2011). Prevalence of topoisomerase I genetic mutations and UGT1A1 polymorphisms associated with irinotecan in individuals of Asian descent. Oncol Lett.

[42] (2018). Las Heras S, Martínez-Balibrea E. CXC family of chemokines as prognostic or predictive biomarkers and possible drug targets in colorectal cancer. World J Gastroenterol.

[43] Heckmann D, Maier P, Laufs S (2013). CXCR4 expression and treatment with SDF-1α or plerixafor modulate proliferation and chemosensitivity of colon cancer cells. Transl Oncol.

[44] Cutler MJ, Lowthers EL, Richard CL, Hajducek DM, Spagnuolo PA, Blay J (2015). Chemotherapeutic agents attenuate CXCL12-mediated migration of colon cancer cells by selecting for CXCR4-negative cells and increasing peptidase CD26. BMC Cancer.

[45] Gongora C, Candeil L, Vezzio N (2008). Altered expression of cell proliferation-related and interferon-stimulated genes in colon cancer cells resistant to SN38. Cancer Biol Ther.

[46] Mustafi R, Dougherty U, Mustafi D (2017). ADAM17 is a tumor promoter and therapeutic target in western diet-associated colon cancer. Clin Cancer Res.

[47] Terashima J, Goto S, Hattori H (2015). CYP1A1 and CYP1A2 expression levels are differentially regulated in three-dimensional spheroids of liver cancer cells compared to two-dimensional monolayer cultures. Drug Metab Pharmacokinet.

[48] D'Angelo E, Natarajan D, Sensi F (2020). Patient-derived scaffolds of colorectal cancer metastases as an organotypic 3D model of the liver metastatic microenvironment. Cancers (Basel).

[49] Fontana F, Carollo E, Melling GE, Carter DRF (2021). Extracellular vesicles: emerging modulators of cancer drug resistance. Cancers (Basel).

[50] Xavier CPR, Caires HR, Barbosa MAG, Bergantim R, Guimarães JE, Vasconcelos MH (2020). The role of extracellular vesicles in the hallmarks of cancer and drug resistance. Cells.

[51] Zhao K, Cheng X, Ye Z (2021). Exosome-mediated transfer of circ_0000338 enhances 5-fluorouracil resistance in colorectal cancer through regulating MicroRNA 217 (miR-217) and miR-485-3p. Mol Cell Biol.

[52] Khorrami S, Zavaran Hosseini A, Mowla SJ, Soleimani M, Rakhshani N, Malekzadeh R (2017). MicroRNA-146a induces immune suppression and drug-resistant colorectal cancer cells. Tumour Biol.

[53] Reya T, Morrison SJ, Clarke MF, Weissman IL (2001). Stem cells, cancer, and cancer stem cells. Nature.

[54] Ricci-Vitiani L, Lombardi DG, Pilozzi E (2007). Identification and expansion of human colon-cancer-initiating cells. Nature.

[55] Fang DD, Kim YJ, Lee CN (2010). Expansion of CD133(+) colon cancer cultures retaining stem cell properties to enable cancer stem cell target discovery. Br J Cancer.

[56] Olszewski U, Liedauer R, Ausch C, Thalhammer T, Hamilton G (2011). Overexpression of CYP3A4 in a COLO 205 colon cancer stem cell model in vitro. Cancers (Basel).

[57] Zhang S, Chatterjee T, Godoy C, Wu L, Liu QJ, Carmon KS (2019). GPR56 drives colorectal tumor growth and promotes drug resistance through upregulation of MDR1 expression via a RhoA-mediated mechanism. Mol Cancer Res.

[58] Planque C, Rajabi F, Grillet F (2016). Pregnane X-receptor promotes stem cell-mediated colon cancer relapse. Oncotarget.

[59] Cunningham D, Pyrhönen S, James RD (1998). Randomised trial of irinotecan plus supportive care versus supportive care alone after fluorouracil failure for patients with metastatic colorectal cancer. Lancet.

[60] Cunningham D, Humblet Y, Siena S (2004). Cetuximab monotherapy and cetuximab plus irinotecan in irinotecan-refractory metastatic colorectal cancer. N Engl J Med.

[61] Masuishi T, Tsuji A, Kotaka M (2020). Phase 2 study of irinotecan plus cetuximab rechallenge as third-line treatment in KRAS wild-type metastatic colorectal cancer: JACCRO CC-08. Br J Cancer.

[62] Cremolini C, Rossini D, Dell'Aquila E (2019). Rechallenge for patients with RAS and BRAF wild-type metastatic colorectal cancer with acquired resistance to first-line cetuximab and irinotecan: a phase 2 single-arm clinical trial. JAMA Oncol.

[63] Pfeiffer P, Yilmaz M, Möller S (2020). TAS-102 with or without bevacizumab in patients with chemorefractory metastatic colorectal cancer: an investigator-initiated, open-label, randomised, phase 2 trial. Lancet Oncol.

[64] Shitara K, Yamanaka T, Denda T (2019). REVERCE: a randomized phase II study of regorafenib followed by cetuximab versus the reverse sequence for previously treated metastatic colorectal cancer patients. Ann Oncol.

[65] Muro K, Boku N, Shimada Y (2010). Irinotecan plus S-1 (IRIS) versus fluorouracil and folinic acid plus irinotecan (FOLFIRI) as second-line chemotherapy for metastatic colorectal cancer: a randomised phase 2/3 non-inferiority study (FIRIS study). Lancet Oncol.

[66] Chu C, Abbara C, Tandia M (2014). Cetuximab increases concentrations of irinotecan and of its active metabolite SN-38 in plasma and tumour of human colorectal carcinoma-bearing mice. Fundam Clin Pharmacol.

[67] Hakata S, Terashima J, Shimoyama Y (2018). Differential sensitization of two human colon cancer cell lines to the antitumor effects of irinotecan combined with 5-aza-2'-deoxycytidine. Oncol Lett.

[68] Shimizu S, Iida S, Ishiguro M (2010). Methylated BNIP3 gene in colorectal cancer prognosis. Oncol Lett.

[69] Burton TR, Gibson SB (2009). The role of Bcl-2 family member BNIP3 in cell death and disease: NIPping at the heels of cell death. Cell Death Differ.

[70] Miyaki Y, Suzuki K, Koizumi K (2012). Identification of a potent epigenetic biomarker for resistance to camptothecin and poor outcome to irinotecan-based chemotherapy in colon cancer. Int J Oncol.

[71] Dos Santos RS, Marroqui L, Velayos T (2019). DEXI, a candidate gene for type 1 diabetes, modulates rat and human pancreatic beta cell inflammation via regulation of the type I IFN/STAT signalling pathway. Diabetologia.

[72] Lee V, Wang J, Zahurak M (2018). A phase I trial of a guadecitabine (SGI-110) and irinotecan in metastatic colorectal cancer patients previously exposed to irinotecan. Clin Cancer Res.

